# Homogeneous liquid–liquid microextraction coupled with HPLC/DAD for determination of nirmatrelvir and ritonavir as COVID-19 combination therapy in human plasma

**DOI:** 10.1186/s13065-023-01080-4

**Published:** 2023-11-24

**Authors:** Inas A. Abdallah, Sherin F. Hammad, Alaa Bedair, Fotouh R. Mansour

**Affiliations:** 1 Department of Analytical Chemistry, Faculty of Pharmacy, University of Sadat City, Sadat City, 32897 Monufia Egypt; 2 Department of Pharmaceutical Analytical Chemistry, Faculty of Pharmacy, Tanta University, Elgeish Street, The Medical Campus of Tanta University, Tanta, 31111 Egypt

**Keywords:** Nirmatrelvir, Ritonavir, COVID-19, HPLC, SARS-CoV-2, Sugaring-out

## Abstract

The study reports the development of a high-performance liquid chromatography/diode array detection method to measure the levels of nirmatrelvir and ritonavir in human plasma. These two antiviral medications are used for the treatment of COVID-19 and are marketed as Paxlovid^®^. The method employed sugaring-out induced homogeneous liquid–liquid microextraction to improve sensitivity. Optimization of the method was performed using the one variable at a time approach by adjusting several factors such as type of sugar, extractant, amount of sugar, volume of extractant, and pH of the aqueous sample to achieve the highest efficiency. The developed method was validated according to the Food and Drug Administration guidelines and demonstrated good linearity, accuracy, and precision. The range of linearity was from 1000 to 20,000 ng/mL for nirmatrelvir and 200 to 20,000 ng/mL for ritonavir with correlation coefficient values of 0.998 and 0.996, respectively. Selectivity studies revealed that no others peaks appeared in the retention times of the studied drugs. The stability of nirmatrelvir and ritonavir were also investigated through short term and three cycles of freeze–thaw, and both drugs were found stable. This analytical method could be useful for monitoring drug concentrations in patients undergoing treatment with these medications for COVID-19. In this work, for the first time, SULLME was used for the sensitive determination of nirmatrelvir and ritonavir in biological fluids. The developed method was able to determine both drugs in therapeutic levels with no need to sophisticated techniques like LC–MS. In addition to that, SULLME is considered a simple and green sample preparation in comparison with conventional sample preparation methods.

**Supplementary Information:**

The online version contains supplementary material available at 10.1186/s13065-023-01080-4.

## Introduction

From the end of 2019 until now, the new coronavirus (COVID-19) has swept the planet. According to the reports released by the World Health Organization (WHO), the global number of persons infected with the virus and the total fatalities had surpassed 750 million and 6.8 million, respectively, as of March, 2023 [1]. Despite the fact that many vaccines have been developed and a significant vaccination rate has been achieved, new variants of this virus continue to emerge due to COVID-19’s ease of mutation, making the development of new drugs, therapeutic strategies, and vaccines critical to controlling the spread of this pandemic [2, 3].

**Fig. 1:**
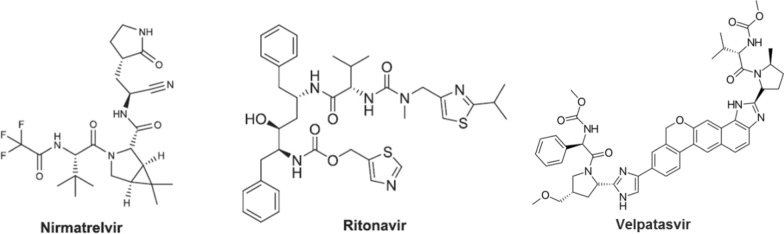
Chemical structures of nirmatrelvir, ritonavir, and velpatasvir

Nirmatrelvir (NIRMA) is a SARS-CoV-2 primary protease inhibitor that prevents viral polyprotein processing and consequently virus multiplication. NIRMA is co-administered with ritonavir (RITONA), a well-known HIV-1 protease inhibitor. RITONA functions primarily as an inhibitor of the enzyme cytochrome P450 3A4 and so avoids early metabolic deactivation of NIRMA [4]. Paxlovid^®^ is a co-formulated antiviral treatment for COVID-19 composed of NIRMA (150 mg) and RITONA (100 mg), whose chemical structures are shown in Fig. 1. The emergency admission usage of Paxlovid^®^ had been approved by the Food and Drug Administration (FDA) for the treatment of adults and children (≥ 12 year and ≥ 40 kg) with mild to severe COVID-19 [5–7]. NIRMA activity had been widely validated in preclinical and phase I clinical trials, the results indicated the use of Paxlovid^®^ would significantly reduce hospitalization and mortality in patients with mild to moderate infection of COVID-19 [7–12]. According to the Food and Drug Administration (FDA) evaluation, the 95th projected NIRMA C_max_ on day 5 of therapy was 10,000 ng/mL in individuals without renal impairment [13], while the C_max_ of RITONA was 11,200 ng/mL [14]. Therapeutic drug monitoring (TDM) of NIRMA and RITONA may boost the safety and effectiveness of Paxlovid^®^ in the treatment of high-risk patient populations [15, 16]. Two LC–MS/MS methods were developed for the simultaneous determination of NIRMA and RITONA in human plasma [17, 18]. However, this sophisticated technique is not available in all analytical laboratories.

Homogeneous liquid–liquid extraction (HLLE) is a type of LLE that uses a water-miscible organic solvent as the extractant [19]. In HLLE, an organic solvent is mixed with an aqueous sample to form a homogeneous phase, which is then separated using a phase separator such as salts [20, 21] or sugar [22, 23]. The contact area between the water aliquot and the extractant is unlimited, and the organic extractant can be analyzed directly without the evaporation/reconstitution step [24, 25], which renders HLLE faster, easier, more efficient specially for polar analytes [26] and more eco-friendly than LLE. Homogeneous liquid–liquid microextraction (HLLME) refers to the use of only a small amount of a water-soluble extractant instead of large volumes of immiscible organic solvents. These small volumes of extractants makes the analyte highly concentrated in the separated organic phase, increasing the sensitivity of the analytical method [27]. In this work, sugaring-out induced homogeneous liquid–liquid microextraction was developed for the determination of NIRMA and RITONA followed by HPLC/DAD. A C8 stationary phase at 35 °C was employed, with a mobile phase consisting of phosphate buffer (50 mM, pH 3) and acetonitrile in volumetric ratio of 35:65, respectively. The developed method was easy, sensitive and suitable for therapeutic drug monitoring of NIRMA and RITONA in human plasma.

## Experimental

### Materials and methods

NIRMA (99.8) and RITONA (99.9%) were kindly supplied by Global Napi Pharmaceuticals (6th of October City, Egypt). Velpatasvir (VTV, 99.9%, internal standard) was kindly obtained from Gilead Sciences (Milano, Italy). Acetonitrile (HPLC grade), potassium dihydrogen phosphate, phosphoric acid, and sodium hydroxide were purchased from Merck (Darmstadt, Germany). Sucrose, fructose, sorbitol, mannitol and tetrahydrofuran, and acetone (analytical grade) were acquired from Alpha Chemicals (Cairo, Egypt). Human plasma samples were kindly provided by Vacsera National Blood Bank (Giza, Egypt).

### Instrumentation

The separations were conducted on a Dionex UltiMate 3000 HPLC (Thermo Scientific™, Dionex™, Sunnyvale, CA, USA). The instrument composed of a WPS-3000TSL autosampler, a LPG-3400SD quaternary pump, a VWD-3000 variable wavelength detector, and a TCC-3000SD column thermostat. Data processing and acquisition were carried out by Chromeleon 7 software. Medilabs Tabletop Centrifuge (Cyan-CL008, Kampala, Uganda) and Jenway® 3510 pH-meter (Staffordshire,UK) were also employed for phase separation and pH adjustment.

### Chromatographic conditions

All chromatographic separations were performed on a Thermo Hypersil ODS C_8_ column (250 × 4.6 mm, 5 μm) at 35 °C. The mobile phase consisted of phosphate buffer (50 mM, pH = 3): acetonitrile (35:65, v/v). The injection volume was 5 µL, and the detector was DAD set at 210 nm. VTV was selected as an internal standard, at a concentration of 20 µg/mL. It is worth indicating that VTV was selected as an internal standard owing to its high structure similarity to the studied antivirals and its reasonable retention time under the optimized chromatographic conditions.

### Standard and working solution preparation

Stock standard solutions of the three drugs were separately prepared at 0.5 mg/mL in methanol and stored at 4 °C until use. Aliquots of these stock solutions were transferred to 25 mL volumetric flask and completed to the mark with deionized water to make aqueous mixtures with a final concentration of 20 μg/mL, each.

### Extraction procedures

In sugaring‐out induced homogeneous liquid‐liquid microextraction (SULLME), 500 μL of ACN was added to a 5 mL screw cap glass test tube containing 1 mL of the aqueous sample followed by vortex for 1 min, then 800 mg sucrose was added followed by vortex for 1 min to dissolve the sugar. The tube was centrifuged for 5 min at 3467*g* (6000 RPM) and the upper layer was transferred for analysis. The procedures were optimized to maximize the peak area which was used to measure of the extraction efficiency. During the SULLME development, each experiment was performed in triplicate.

### Method validation

The HPLC method validation was performed according to the US Food and Drug Administration for bioanalytical method validation guidelines (FDA) [28] with respect to selectivity, linearity and range, limit of quantitation (LOQ), accuracy, precision and stability.

#### Selectivity

The method’s selectivity was assessed by evaluating human plasma from six distinct sources to look for possible interferences, with NIRMA and RITONA peaks. Blank plasma samples were created (without analytes) and chromatographically compared to another set of standard samples spiked with NIRMA and RITONA at their respective concentration.

#### Linearity and range

The calibration curve was created by plotting the nominal standard concentration against the peak area ratio of NIRMA and RITONA to VTV. The selected concentrations of NIRMA were 1000, 2000, 3000, 5000, 15,000 and 20,000 ng/mL, while the selected concentrations of RITONA were 200, 500, 1000, 2000, 3000, 5000, 10,000, 15,000 and 20,000 ng/mL, the concentration of VTV was 25,000 ng/mL.

#### Accuracy and precision

The accuracy and the intra-day precision were assessed by analyzing six replicates containing NIRMA and RITONA at four quality control (QC) levels: LLOQ, low QC (LQC), medium QC (MQC), and high QC (HQC), which were1000, 3000, 10,000, 18,000 ng/ml for NIRMA and 200, 600, 6000 and 18,000 ng/mL for RITONA. Inter-day accuracy and precision were determined by assessing six replicates containing NIRMA and RITONA at four QC samples on three separate days. The proposed method’s accuracy was evaluated as a % recovery. The FDA guidelines indicated that the recovery (%) should not exceed 15% for all QC levels except the LLOQ, which is allowed to be 20% or less of the nominal values. The relative standard deviation RSD (%) was used to assess precision. The acceptable standards for RSD (%) are 15% across the QC samples except that 20% at the LLOQ is allowed.

#### Stability

The stability of NIRMA and RITONA in human plasma was studied at various storage conditions including benchtop and freeze–thaw. The benchtop stability testing was done after keeping the sample at room temperature for 4 h. The freeze–thaw stability investigation was carried out in three cycles. At each cycle, samples were frozen for 12 h before being examined to determine the stability of NIRMA and RITONA under various circumstances. The results were then compared to samples that had been newly prepared. If the RSD (%) was less than 15% when compared to newly prepared samples, the samples were stable.

#### Application to biological samples

**Fig. 2:**
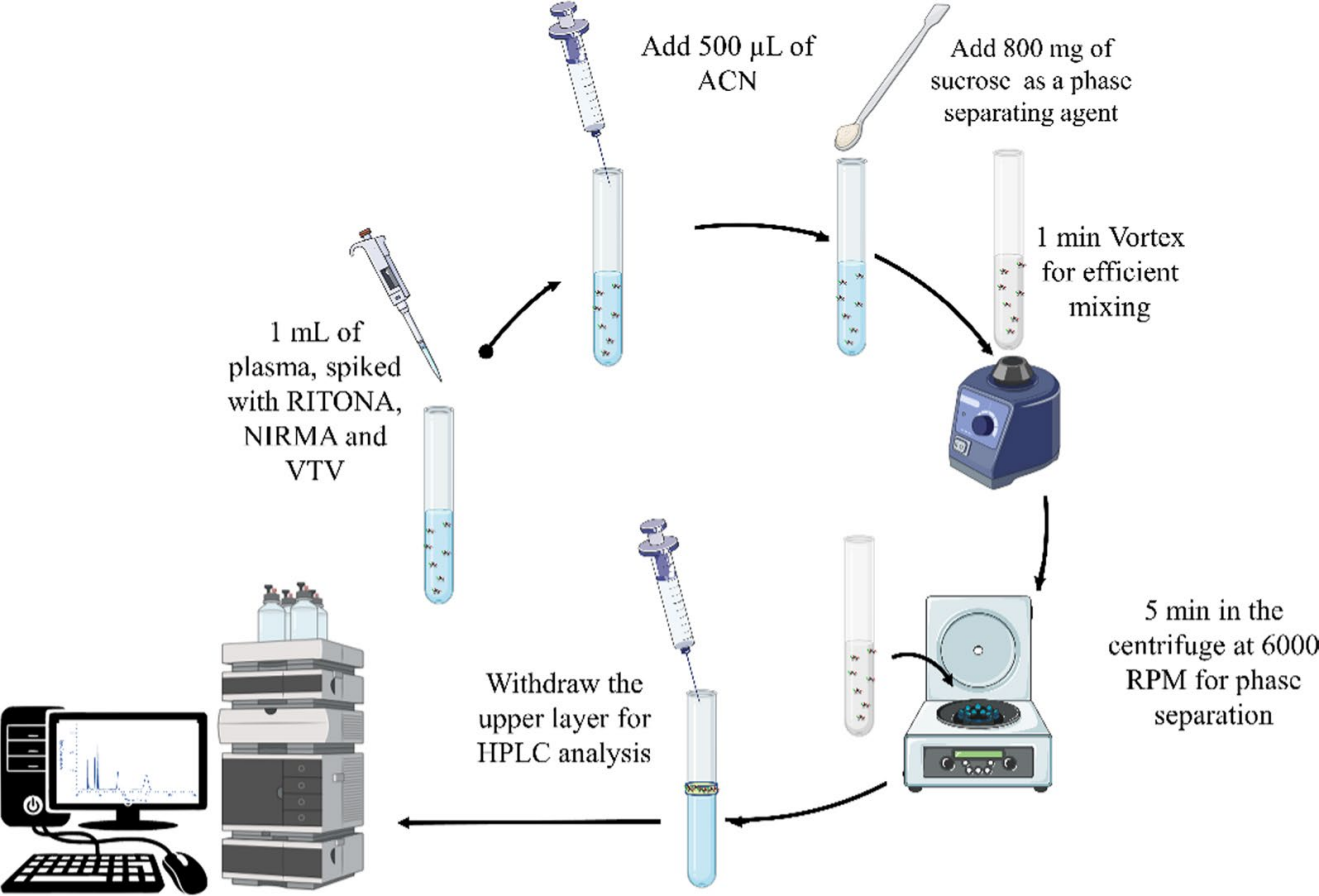
Procedures of the determination of NIRMA and RITONA by SULLME followed by HPLC/DAD

The SULLME procedures were carried out as follows: 1 mL of the plasma sample, spiked with VTV (20 µg/mL), NIRMA, and RITONA at the desired concentrations was vortexed for 1 min after pH adjustment to 4. Then, 500 µL of acetonitrile was added to the sample, and the tube was vortexed for another minute. Next, 800 mg of sucrose was added, and the mixture was vortexed again for 1 min before centrifugation at 6000 rpm for 5 min to induce phase separation by sugaring out. The upper layer was pipetted and transferred into HPLC vials for analysis. Figure 2 illustrates the procedure performed during SULLME using 800 mg of sucrose as a phase separating agent.

## Results and discussion

**Fig. 3:**
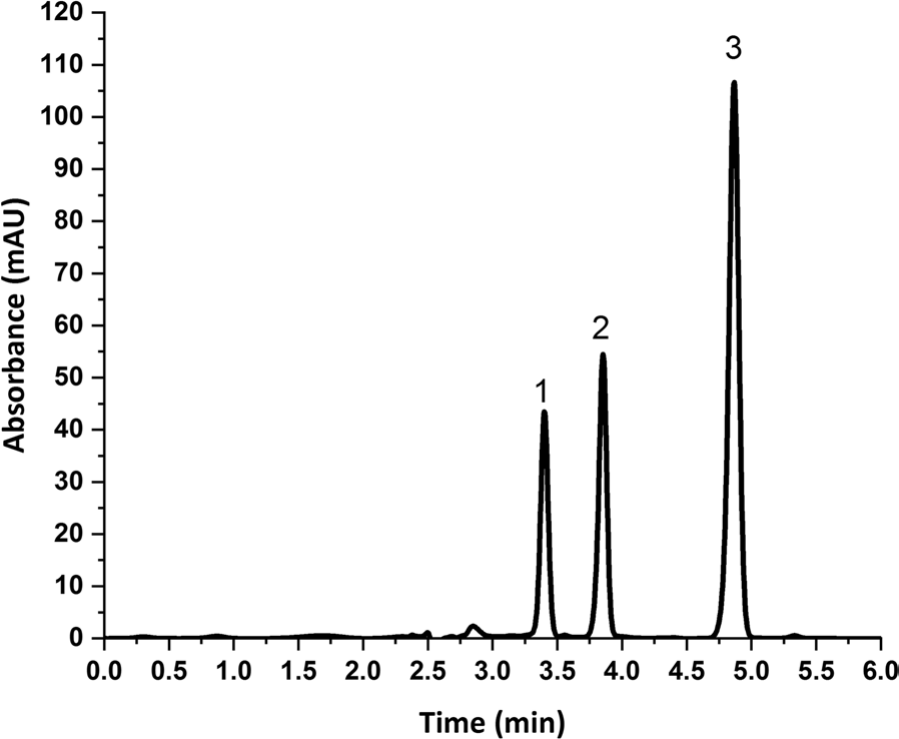
Chromatographic separation of **1** velpatasvir (IS, 20 µg/mL), **2** nirmatrelvir (20 µg/mL) and **3** ritonavir (20 µg/mL) in an aqueous sample. Chromatographic conditions: Column: Thermo Hypersil ODS C_8_ column (250 × 4.6 mm, 5 μm) at 35 °C, Mobile phase: phosphate buffer (50 mM, pH = 3): acetonitrile (35:65, v/v), Elution: Isocratic, Detection: DAD at 210 nm, Flow rate: 1 mL/min, Injection volume: 5 µL

To develop the chromatographic separation of the three antiviral drugs, the pH of the mobile phase was investigated together with the ratio between the aqueous buffer and the organic modifier. The pKa values of the studied drugs were 7.1 (acidic) and − 1.6 (basic) for NIRMA and 13.68 (acidic) and 2.84 (basic) for RITONA. Accordingly, pH values in the range 2.8–6.1 were selected for the mobile phase buffer to guarantee that both drugs are predominately in the unionized form. Acceptable resolution was obtained using phosphate buffer (50 mM, pH 3): ACN (45:55, v/v), but the peak of NIRMA was fronted (As = 0.73), as shown in Additional file 1: Fig. S1. No significant improvements in peak shapes were obtained by changing the buffer pH. Increasing the percentage of the organic modifier in the mobile phase from 55 to 65% improved the peak symmetry. Higher percentages of ACN induced peak overlap between VTV and NIRMA. So, a mobile phase consisting of phosphate buffer (50 mM, pH 3): ACN (35:65, v/v) was selected for the separation of this mixture. Figure 3, shows the chromatographic separation of the three drugs in aqueous samples at the optimum conditions.

### Method evaluation

Different experimental variables of SULLME were studied to achieve the maximum enrichment. These variables included the type and volume of extracting solvents, the type and amount of sugar and the sample pH. Optimization of these parameters was performed using the one-variable-at-a-time approach, by monitoring the peak areas at each condition.

#### Organic solvent and sugar type optimization

**Fig. 4:**
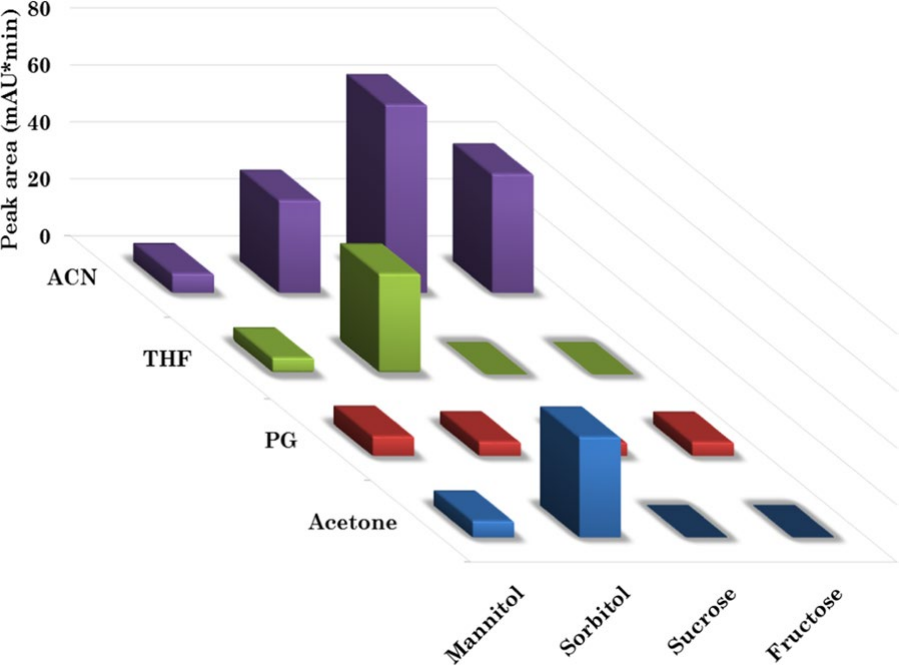
Effect of sugar and extracting solvent types on the microextraction efficiency of RITONA

Different water miscible organic solvents were investigated as extractants including ACN, acetone, THF and propylene glycol. Four sugars (mannitol, sorbitol, sucrose and fructose) were tried as phase separating agents with each extractant. The tube was vortexed for 1 min to ensure complete dissolving of sugars, then the tube was centrifuged at 6000 rpm for 5 min for complete phase separation. Distinct phase separation was observed when ACN, acetone and THF were employed as extractant, in presence of sorbitol, sucrose and fructose as phase separating agents. The optimum extractant/phase separating agent combination was selected based on the intensities of the RITONA and NIRMA peaks. As revealed in Fig. 4, ACN/sucrose achieved the best microextraction efficiency for RITONA. Similar results were observed for NIRMA (Additional file 1: Fig. S2). Accordingly, ACN was selected as the best extractant, using sucrose as a phase separating agent in the following procedure.

#### ACN volume optimization

**Fig. 5:**
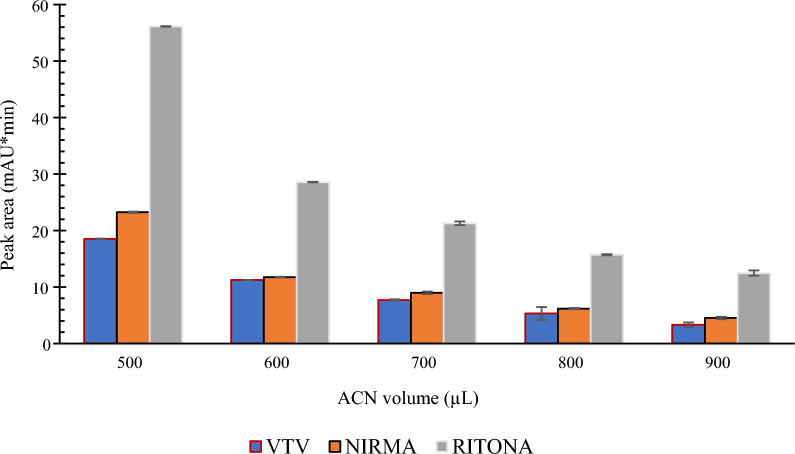
The effect of ACN volume on the extraction efficiency using 1 mL aqueous sample and 1000 mg of sucrose as a phase separating agent

The extractant volume is the most important experimental parameter that could affect the sample enrichment in SULLME. Generally, analyte pre-concentration is inversely proportional to the volume of extractant. Different volumes of ACN were investigated in the range of 500 to 1000 µL. As shown in Fig. 5, the highest response was observed using 500 μL ACN, thus it was designated as the optimum extractant volume. It is worth mentioning that 500 µL was the lowest possible volume to observe phase separation between the aqueous sample and ACN after sugaring out by sucrose. Using volumes of acetonitrile lower than 500 μL could not induce definite phase separation.

#### Sucrose amount optimization

**Fig. 6:**
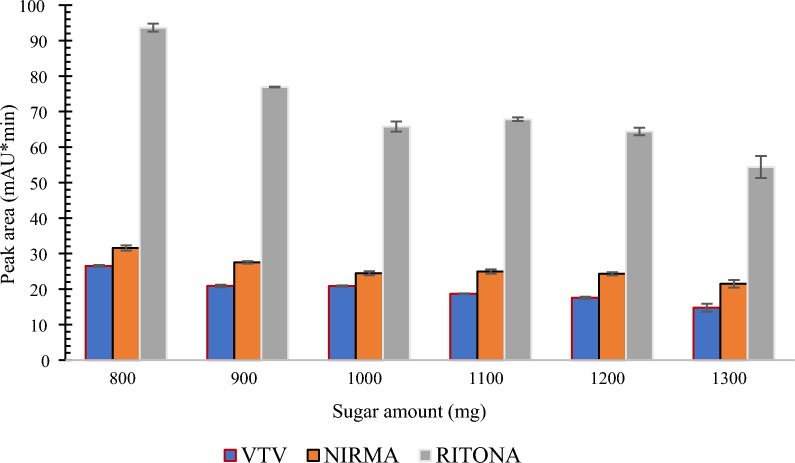
The effect of sucrose amount on the extraction efficiency using 1 mL aqueous sample and 500 µL of ACN

The sugar amount is another variable that could affect the efficiency in SULLME. Very small amounts of sugars may fail to induce phase separation, while larger than needed amounts would be a waste of resources and could deteriorate the method performance. Sucrose amount was inversely proportional on the extraction efficiency. An amount of 800 mg of sucrose was the practical limit to induce phase separation. As shown in Fig. 6, the highest peak areas were observed when 800 mg of sucrose were employed, therefore this amount was added in the following procedure.

#### pH optimization

**Fig. 7:**
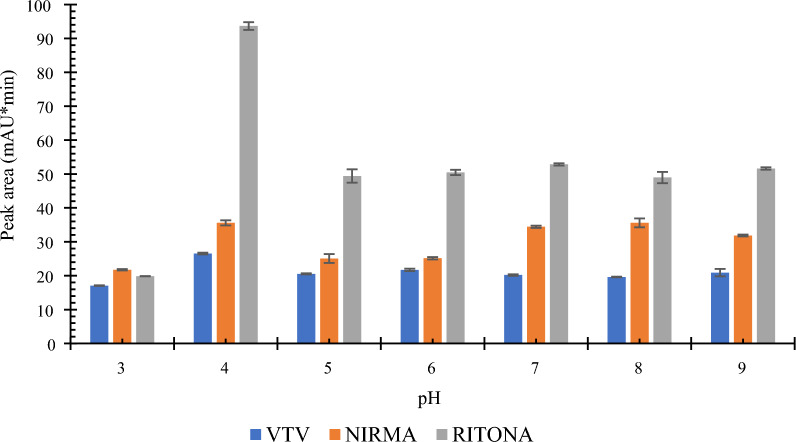
The effect of pH of the aqueous sample on the extraction efficiency using 1 mL aqueous sample, 500 µL of ACN and 800 mg of sucrose

In extraction methods, the pH of the aqueous sample has a critical role because of its effect on the ionization and solubility of drugs. Different values of pH were studied in the range of 3 to 9. Acidic pH values were adjusted by 10% phosphoric acid, while alkaline pH values were adjusted by sodium hydroxide. As indicated in Fig. 7**,** the maximum extraction efficiency was achieved at pH 4 for all antiviral drugs. This high extraction efficiency at pH 4 could be due to the predominance of unionized forms of the analytes. NIRMA has two pKa values, 7.1 (acidic) and − 1.6 (basic). At pH 4, NIRMA will be in the unionized form, which facilitates its transfer to the organic extractant and improve extraction efficiency. Similarly, RITONA has two pKas, 13.68 (acidic) and 2.84 (basic). At pH 4, RITONA will also be predominantly in the neutral form, which explains the high peak intensity. The effect of pH on VTV was also studied, just to guarantee that the selected conditions are suitable for the internal standard. VTV has an acidic pKa at 11.14 and a basic pKa at 5.97. Although higher peak intensities of VTV were obtained in the basic pH side, pH = 4 was selected as the optimum pH because the objective was to increase the peak intensities of NIRMA and RITONA, while using VTV as an internal standard to correct for microextraction errors.

### Method validation

#### Selectivity

**Fig. 8:**
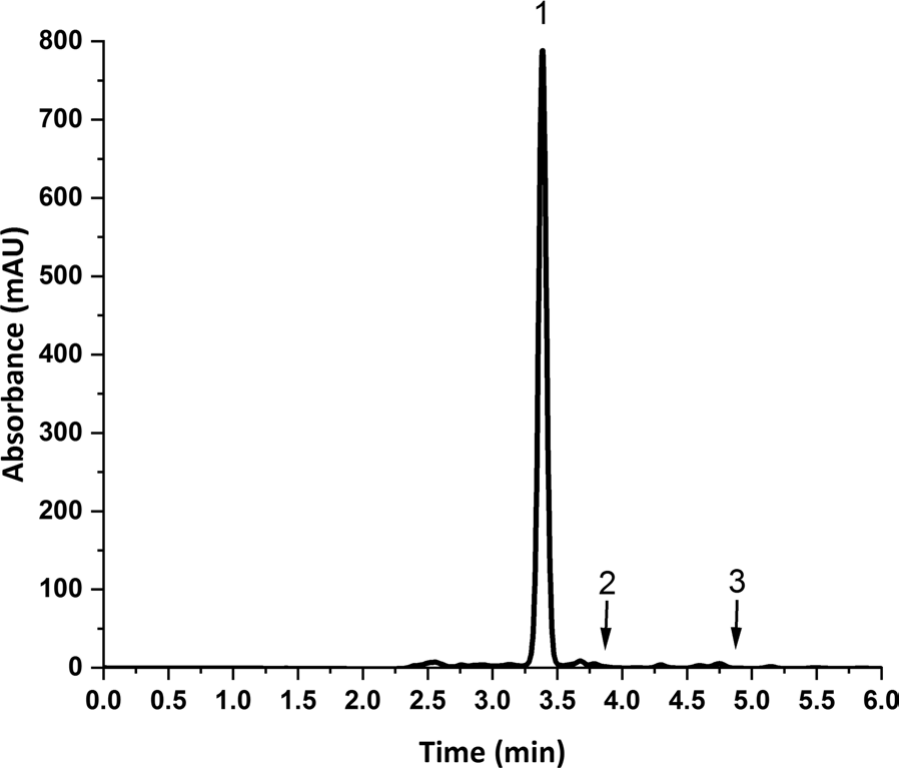
Blank plasma sample spiked with velpatasvir (**1**, 20 µg/mL) as an internal standard. The arrows show the absence of peaks at the retention times of nirmatrelvir (**2**) and ritonavir (**3**). Chromatographic conditions: Column: Thermo Hypersil ODS C_8_ column (250 × 4.6 mm, 5 μm) at 35 °C, Mobile phase: phosphate buffer (50 mM, pH = 3): acetonitrile (35:65, v/v), Elution: Isocratic, Detection: DAD at 210 nm, Flow rate: 1 mL/min, Injection volume: 5 µL

The selectivity of the SULLME was studied by analyzing six individual human blank plasma. The chromatograms of each blank plasma, spiked with the internal standard only was compared with human plasma samples containing NIRMA, RITONA and VTV to test for the presence of any interferences. The chromatograms showed no interfering peaks at the retention times of NIRMA nor RITONA as indicated in Fig. 8, and the %interferences were calculated at the LOQ and were found to be 0.67 and 0.39% for NIRMA, RITONA, respectively which indicates the selectivity of the developed method.

#### Linearity, range and limit of quantitation

The calibration curve was created by plotting peak area ratios (NIRMA and RITONA to VTV) versus NIRMA and RITONA concentrations. The linearity range was determined to be 1000–20,000 for NIRMA with a correlation value of 0.9978, while the linearity range for RITONA was 200–20000 ng/with a correlation coefficient of 0.996. The ANOVA lack-of-fit test has been performed for both calibration curves at 95% confidence interval, and no significant lack of fit was observed, indicating acceptable linearities. The quantitation limit was established by identifying the lowest concentration of NIRMA and RITONA in human plasma that can be quantified reliably and precisely.

#### Accuracy and precision

Accuracy and precision were assessed at four levels of QC samples, including LLOQ, LQC, MQC, and HQC, each of which was tested in sextuplicate. The accuracy was assessed using the %recovery, while repeatability and intermediate precision were assessed using the RSDs (%) within-day and between-days, respectively. As indicated in Table 1, % recovery ranged between 89.48 and 105.60, which is deemed acceptable according to the FDA guidelines where % RSDs were in the range 0.33–13.05%, which is acceptable according to FDA guidelines. These results indicate that the method is adequately accurate and sufficiently precise for the TDM of both drugs in human plasma.

**Table 1:** Accuracy intraday and interday and precision of quality control concentrations

	QC concentration	Intraday			Interday		
		Added (ng/mL)	Found (ng/mL)	Found (%) ± RSD	Added (ng/mL)	Found (ng/mL)	Found (%) ± RSD
NIRMA	LLOQ	1000	1013	101.27 ± 4.86	1000	1005	100.51 ± 0.98
	LQC	3000	3129	104.30 ± 7.15	3000	3168	105.60 ± 5.47
	MQC	10,000	9688	96.88 ± 10.92	10,000	10,250	102.50 ± 6.79
	HQC	18,000	17,676	98.20 ± 1.93	18,000	18,762	104.23 ± 6.33
Mean				100.16			103.21
% RSD				6.22			4.891
RITONA	LLOQ	200	179	89.48 ± 13.05	200	182	91.02 ± 11.08
	LQC	6001	580	96.61 ± 11.25	6001	631	105.22 ± 7.09
	MQC	6000	5778	96.30 ± 11.28	6000	5565	92.76 ± 4.87
	HQC	18,000	17,972	99.85 ± 0.33	18,000	18,038	100.21 ± 1.64
Mean				95.56			97.30
%RSD				8.98			3.77

#### Stability studies

The stability of NIRMA and RITONA were investigated in spiked plasma samples, through processing and storage conditions. QCL and QCH were used to study short term and 3 cycles of freeze and thaw, the results were compared with the initial concentrations of freshly prepared samples. Table 2 indicated the results of stability studies of NIRMA and RITONA under benchtop and freeze/thaw conditions. The QCL and QCH were within the range of ± 15% which are acceptable by the FDA guidelines. These values of %RSD prove that NIRMA and RITONA were stable for routine analysis and at three cycles of freeze and thaw.

**Table 2:** Results of the benchtop and the freeze/thaw stability studies

	Amount added (ng/mL)	Amount found (ng/mL)	% Found ± RSD
Bench top			
NIRMA	3000	3345	111.51 ± 6.42
	18,000	20,263	112.57 ± 6.24
RITONA	600	663	110.54 ± 4.81
	18,000	18,711	103.95 ± 0.611
Freeze and thaw 1st Cycle			
NIRMA	3000	3302	110.08 ± 9.26
	18,000	19,930	110.72 ± 2.68
RITONA	600	606	100.93 ± 6.29
	18,000	19,667	109.26 ± 1.55
Freeze and thaw 2nd cycle			
NIRMA	3000	3344	111.47 ± 6.67
	18,000	18,258	101.44 ± 7.96
RITONA	600	683	113.87 ± 4.1
	18,000	17,696	98.31 ± 7.48
Freeze and thaw 3rd cycle			
NIRMA	3000	3239	107.97 ± 4.87
	18,000	20,561	114.23 ± 5.13
RITONA	600	681	113.45 ± 7.52
	18,000	19,540	108.58 ± 1.89

### Application of the developed method to human plasma

To investigate the applicability of the proposed method on human plasma, three different plasma lots were examined at two concentration levels (5000 and 15,000 ng/mL) as listed in Table 3. After performing sample preparation procedures as indicated in the extraction procedures in the Experimental Section, the upper layer of ACN was then pipetted and transferred to an HPLC vial for analysis. As shown in Tables 3, the % recovery was is the range 91.75–109.12, and the % RSD was ≤ 5.09 Thus, the developed method is applicable to human plasma, according to the acceptance criteria of the FDA regulations for bioanalytical methods.

**Table 3:** Application of the developed HPLC method on different plasma samples

	Amount added (ng/mL)	Amount found (ng/mL)	%Found ± RSD
Plasma 1			
NIRMA	5000	5241	104.82 ± 5.09
	15,000	14,748	98.32 ± 1.23
RITONA	5000	5177	103.53 ± 3.37
	15,000	15,537	103.58 ± 4.12
Plasma 2			
NIRMA	5000	4910	98.19 ± 3.12
	15,000	16,368	109.12 ± 2.98
RITONA	5000	4995	99.90 ± 3.67
	15,000	15,923	106.15 ± 1.4
Plasma 3			
NIRMA	5000	4588	91.75 ± 2.16
	15,000	14,373	95.82 ± 3.41
RITONA	5000	5359	107.18 ± 4.58
	15,000	14,703	98.02 ± 4.91

### Comparison with other reported methods

**Fig. 9:**
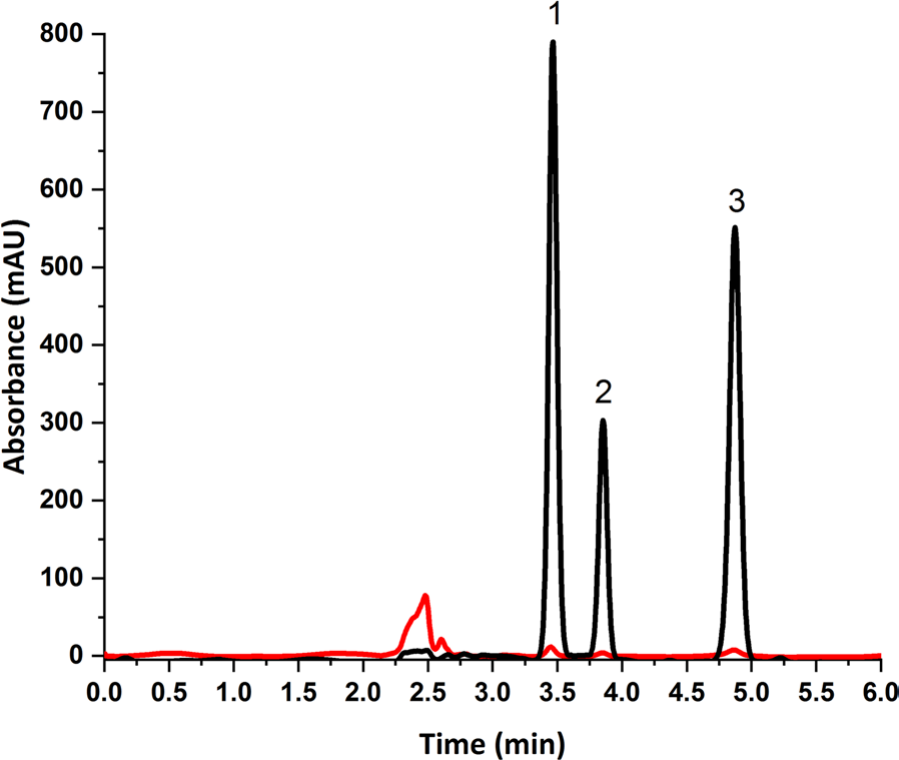
Chromatograms of human plasma containing **1** velpatasvir (IS, 20 µg/mL), **2** nirmatrelvir (15 µg/mL) and **3** ritonavir (15 µg/mL) after sample preparation by protein precipitation (red) and SULLME (black). Chromatographic conditions: Column: Thermo Hypersil ODS C_8_ column (250 × 4.6 mm, 5 μm) at 35 °C, Mobile phase: phosphate buffer (50 mM, pH = 3): acetonitrile (35:65, v/v), Elution: Isocratic, Detection: DAD at 210 nm, Flow rate: 1 mL/min, Injection volume: 5 µL

A few reported methods for the determination of NIRMA and RITONA in different matrices have been developed (Table 4). To the best of our knowledge, just one HPLC/UV method has been reported for the determination of NIRMA and RITONA in dosage forms [29], but the method applicability to plasma samples is questionable due to the limited sensitivity. On the other hand, two LC–MS/MS method could simultaneously measure NIRMA and RITONA in biological matrices [17, 18]. However, both method employed protein precipitation during sample preparation. The dilution effect of protein precipitation makes it not possible to use this sample preparation approach before HPLC/UV analysis. As indicated in Fig. 9, SULLME achieved substantially higher sensitivity in comparison with the reported protein precipitation method [17], under the same chromatographic conditions. The developed HPLC/UV method is sensitive enough for real plasma sample analysis with sufficient accuracy and precision. Furthermore, SULLME approach is more environmentally friendly, simpler, and more efficient than protein precipitation due to sample enrichment.

**Table 4:** The reported chromatographic methods for determination of NIRMA and RITONA

Sample	Sample preparation	Detection technique	Linearity (ng/mL.)	% RSD	LOQ	Refs.
Plasma	Protein precipitation	LC–MS/MS	10–10,000 ng/mL for nirmatrelvir and 2–2000 ng/mL for ritonavir	≤ 13.6	20 and 4 ng/mL for nirmatrelvir and ritonavir respectively	[18]
Plasma	Protein precipitation	LC–MS/MS	50–5000 ng/mL for nirmatrelvir and 10–1000 ng/mL for ritonavir	≤ 14.9	100.0 and 20.0 ng/mL for nirmatrelvir and ritonavir respectively	[17]
Pharmaceutical preparation and plasma	Protein precipitation	TLC	10–50 ng/band	≤ 0.982	2.106 and 1.304 ng/band for nirmatrelvir and ritonavir respectively	[30]
Pharmaceutical preparation	N/A	HPLC–UV/VIS	1000–20,000 ng/mL for both drugs	≤ 0.501	0.60 and 0.96 µg/mL	[29]
Plasma	SULLME	HPLC–DAD	1000 to 20,000 ng/mL for nirmatrelvir and 200 to 20,000 ng/mL for ritonavir	≤ 13.05	3 and 0.6 µg/mL	This work

## Conclusion

In this work, the proposed method can measure NIRMA and RITONA at the same time in patients using Paxlovid^®^ for COVID-19 therapy. The selectivity of the HPLC–DAD detection was adequate to exclude all interferences. In addition, good linearity, acceptable accuracy and precision were achieved according to the FDA guidelines. Both analytes remained stable during the analysis and for at least there freeze and thaw cycles. The approach is useful in supporting TDM and thereby increasing the safety and efficacy of Paxlovid^®^ therapy. Patients who are at high risk of developing severe illness following COVID-19 infections but are currently advised not to use Paxlovid^®^ due to the challenging TDM may benefit from this approach. However, the developed method was tested on spiked samples. Moreover, trying this method in special populations such as lipemic and hemolyzed plasma requires further investigations. Applying this method in clinical studies on real samples will be a good extension of this work, to study the pharmacokinetics or the potential drug-drug or drug-food interactions.

### Supplementary Information

**Additional file 1:** **Figure S1**. Chromatographic separation of nirmatrelvir (20 µg/mL) in an aqueous sample showing the peak fronting. Chromatographic conditions: Column: Thermo Hypersil ODS C_8_ column (250 × 4.6 mm, 5 μm) at 35 °C, Mobile phase: phosphate buffer (50 mM, pH = 3): acetonitrile (45:55, v/v), Elution: Isocratic, Detection: DAD at 210 nm, Flow rate: 1 mL/min, Injection volume: 5 µL. **Figure S2.** Effect of sugar and extracting solvent types on the microextraction efficiency of NIRMA.

## Data Availability

Data are available on reasonable request.

## Abbreviations

HLLE: Homogeneous liquid–liquid extraction
HLLME: Homogeneous liquid–liquid microextraction
LLE: Liquid–liquid extraction
NIRMA: Nirmatrelvir
RITONA: Ritonavir
SULLME: Sugaring-out induced liquid–liquid microextraction
TDM: Therapeutic drug monitoring
