# Supplementary material for: Homogeneous liquid–liquid microextraction coupled with HPLC/DAD for determination of nirmatrelvir and ritonavir as COVID-19 combination therapy in human plasma
Source: BMC Chem. 2023 Nov 24;17(1):166. doi: 10.1186/s13065-023-01080-4 (PMC10675862; doi:10.1186/s13065-023-01080-4)
Supplement: Supplementary file 1 — Additional file 1: Figure S1. Chromatographic separation of nirmatrelvir (20 µg/mL) in an aqueous sample showing the peak fronting. Chromatographic conditions: Column: Thermo Hypersil ODS C8 column (250 × 4.6 mm, 5 μm) at 35 °C, Mobile phase: phosphate buffer (50 mM, pH = 3): acetonitrile (45:55, v/v), Elution: Isocratic, Detection: DAD at 210 nm, Flow rate: 1 mL/min, Injection volume: 5 µL. Figure S2. Effect of sugar and extracting solvent types on the microextraction efficiency of NIRMA. [file 13065_2023_1080_MOESM1_ESM.docx]

**Homogeneous Liquid-Liquid Microextraction Coupled with HPLC/DAD for Determination of Nirmatrelvir and Ritonavir as COVID-19 Combination Therapy in Human Plasma**

Inas A. Abdallah ^1^, Sherin F. Hammad^2^, Alaa Bedair^1^, Fotouh R. Mansour^2^

*^1^ Department of Analytical Chemistry, Faculty of Pharmacy, University of Sadat City, Sadat City, Egypt*

*^2^ Department of Pharmaceutical Analytical Chemistry, Faculty of Pharmacy, Tanta University, Tanta, Egypt*

**Supplementary materials**

**
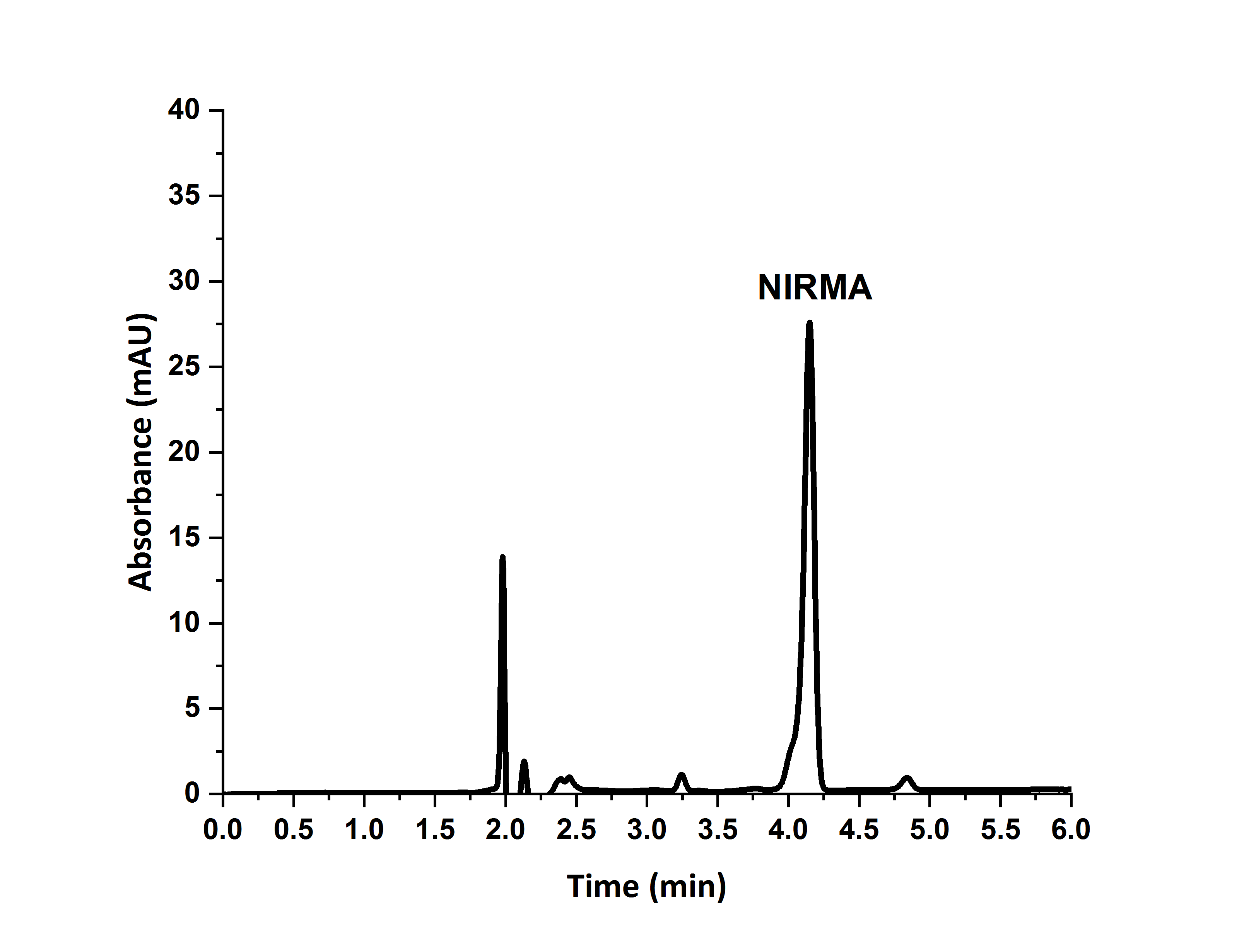
**

**Figure S1**. Chromatographic separation of nirmatrelvir (20 µg/mL) in an aqueous sample showing the peak fronting. Chromatographic conditions: Column: Thermo Hypersil ODS C_8_ column (250 × 4.6 mm, 5 μm) at 35˚C, Mobile phase: phosphate buffer (50 mM, pH = 3): acetonitrile (45:55, v/v), Elution: Isocratic, Detection: DAD at 210 nm, Flow rate: 1 mL/min, Injection volume: 5 µL.

**
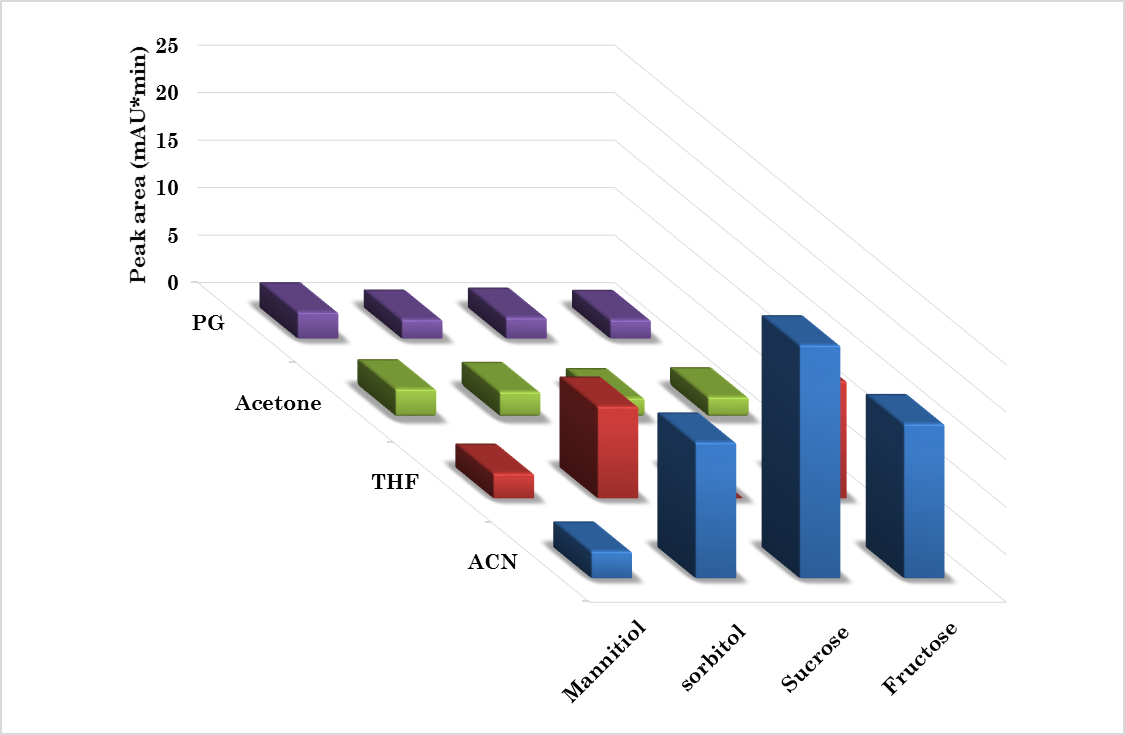
**

**Figure S2:** Effect of sugar and extracting solvent types on the microextraction efficiency of NIRMA.
